# Musical Engagement and Parent-Child Attachment in Families With Young Children During the Covid-19 Pandemic

**DOI:** 10.3389/fpsyg.2021.641733

**Published:** 2021-03-22

**Authors:** Selena Steinberg, Talia Liu, Miriam D. Lense

**Affiliations:** ^1^Department of Psychology and Human Development, Vanderbilt University, Nashville, TN, United States; ^2^Department of Otolaryngology – Head and Neck Surgery, Vanderbilt University Medical Center, Nashville, TN, United States; ^3^Department of Hearing and Speech Sciences, Vanderbilt University, Nashville, TN, United States

**Keywords:** music, Covid-19, parent-child attachment, parent-child interaction, early childhood

## Abstract

The onset of the Covid-19 pandemic has disrupted the lives of families in the United States and across the world, impacting parent mental health and stress, and in turn, the parent-child relationship. Music is a common parent-child activity and has been found to positively impact relationships, but little is known about music’s role in parent-child interactions during a pandemic. The current study utilized an online questionnaire to assess the use of music in the home of young children and their parents in the United States and Canada during Covid-19 and its relationship with parents’ affective attachment with their child. Musical activity was high for both parents and children. Parents reported using music for both emotion regulation and to socially connect with their children. Parent-child musical engagement was associated with parent-child attachment, controlling for relevant parent variables including parent distress, efficacy, education, and parent-child engagement in non-musical activities. These results indicate that music may be an effective tool for building and maintaining parent-child relationships during a period of uncertainty and change.

## Introduction

“During times of sadness or stress we listen to favorite songs, dance, and this relieves stress and lifts moods”

– parent of a 67 month old.

“My youngest son pretty much lives through music, and it seems to be more acute now than before the lockdown”

– parent of a 33 month old.

The unprecedented Covid-19 global pandemic has impacted families’ daily lives across the world. A record number of Americans filed for unemployment in light of the pandemic, with the unemployment rate rising from 3.5% in February 2020 to 14.7% in April 2020, a rate higher than it had been in 80 years (Altig et al., 2020). In particular, the pandemic has been associated with an increase in risk factors for many mental health conditions as a result of social distancing regulations and state-wide stay-at-home orders. According to a Center for Disease Control report from August 2020, 40.9% of over 5,000 United States adults reported at least one adverse mental or behavioral health condition (Czeisler et al., 2020). Compared to reported symptoms in 2019, the prevalence of symptoms of anxiety disorder more than tripled and the prevalence of depressive disorder nearly quadrupled (Czeisler et al., 2020).

In addition to the impact of the Covid-19 pandemic on the stress levels and mental health of individuals, the pandemic rapidly altered family life. Many schools and child care centers (e.g., daycares) closed and parent caregivers lost wages and jobs or had to work remotely (Bartik et al., 2020; U.S. Chamber of Commerce Foundation, 2020). Caregivers had to care for and entertain their children and simultaneously support their families financially and keep them safe. Working mothers of children under 5 years old reported increased difficulty balancing work and family during Covid-19 (Del Boca et al., 2020). During the pandemic, parents spent more time with their children in different types of activities, including playing more games, watching more television, and playing with more toys (Lee et al., 2020). Because of the added stressors of parenting during a pandemic, caregivers of children were at an increased risk of experiencing adverse mental health symptoms compared to non-parents (Russell et al., 2020). Parents’ reports of daily mood were significantly more negative during the pandemic than before its onset (Gassman-Pines et al., 2020). Furthermore, the additional parenting needs and increased stressors were contributors to compromised parent-child relationships (Russell et al., 2020). In a national survey study assessing the physical and emotional well-being of parents and children in the United States in June 2020, there were significant links between parents’ mental health, children’s stress, and parent-child closeness and conflict (Russell et al., 2020). Parents who greatly increased time spent with their child due to the pandemic experienced higher levels of stress, anxiety, and frustration toward their child (Calarco et al., 2020). In light of Covid-19’s impact on family life, it is important to consider activities that parents’ may use to regulate their mental health/emotional state and their connection with their children.

Music can positively impact mental health in both clinical and non-clinical populations. Group singing can serve as a coping mechanism for people experiencing adverse situations (Von Lob et al., 2010). Music therapy may be an effective treatment for depression (Hanser and Thompson, 1994; Erkkilä et al., 2011) and anxiety (Bulfone et al., 2009) in a variety of populations. Regarding parents specifically, after participating in a ten-week group music therapy program, parents facing disadvantages or with a child with a disability reported improvements in their mental health and decreased levels of irritability (Nicholson et al., 2008). Participation in a group parent-infant music program was associated with faster improvements in mothers’ postnatal depressive symptoms vs. participation in a non-music play group or usual care (Fancourt and Perkins, 2018a; Perkins et al., 2018).

Furthermore, everyday musical experiences aid in emotion regulation (Thayer et al., 1994) and stress reduction (Yehuda, 2011). In everyday situations, people select what music to listen to based upon their current emotional situation; in other words, listeners seek out music that can control, support, or change their emotional state (Thoma et al., 2012). Listening to music is associated with lower subjective stress levels (Linnemann et al., 2015) and with lower levels of cortisol (Kreutz et al., 2012). In particular, maternal singing to babies and infants has been found to modulate parents’ and infants’ arousal levels (Cirelli et al., 2020). Additionally, mothers smile more when singing than speaking to their infant (Trehub et al., 2016) and report higher positive affect following singing with their infant as compared to non-musical play (Fancourt and Perkins, 2018b). In a survey of over 2,000 parents with children under 3 years of age, parents who sang or played music daily reported fewer depressive symptoms than those that did not (Custodero et al., 2003).

Music also supports connectedness to others and social bonding (Freeman, 2000; Dunbar, 2012). Parents frequently report using music with their baby as a way to encourage interaction and connectedness (Custodero and Johnson-Green, 2008). The mother-baby bond was significantly stronger for mothers who were encouraged to sing to their babies compared to mothers that were not (Persico et al., 2017). Parents participating in shared parent-child musical experiences report increased closeness to their child compared to those participating in non-musical play (Fancourt and Perkins, 2018b). Even during early childhood, joint musical experiences are associated with children’s prosocial behavior toward other children or adults (Kirschner and Tomasello, 2010; Cirelli et al., 2014, 2018).

Several particular elements of music may support its relationship to social bonding. Tarr et al. (2014) suggest that two mechanisms that support the relationship between music and social bonding are interpersonal synchronization and the release of endorphins. Music has a predictable, rhythmic structure that facilitates interpersonal movement synchrony with others (Savage et al., 2020). The pitch range and scale structure of children’s music may also allow for coordinating vocal interactions, supporting group experiences and connectedness (Savage et al., 2020). Additionally, the repetitive structure and multimodal social cues (voice, facial expressions, movement, and touch) employed during musical games sustain children’s attention and support their social learning particularly when in the meaningful context of parent-child interactions (Lense and Jones, 2016; Mehr et al., 2016; Trehub et al., 2016).

Musical experiences are natural and common in early childhood interactions (Politimou et al., 2018). Several studies focused on characterizing the musical experiences of infants reported frequent musical activity and exposure (e.g., Custodero et al., 2003; Mendoza and Fausey, 2019). Studies of preschool children are rarer and have mixed results, with some reporting the use of music daily (Blackburn, 2017) and others reporting less frequent use (de Vries, 2009). The types of musical activities in early childhood are also varied (Steinberg et al., 2020b). Several studies note that singing is a frequent parent-child musical activity (Ilari, 2005; Blackburn, 2017; Mendoza and Fausey, 2019). However, other common children’s musical activities include the use of musical toys (Mendoza and Fausey, 2019), playing recorded music (de Vries, 2009; Mendoza and Fausey, 2019), and listening and dancing to music with others (Blackburn, 2017). Different music activities can have different purposes. For instance, parents may use passive musical activities, like listening to music or watching music videos, to keep their child occupied, while active, joint parent-child music activities, like singing or dancing, may be used as a social experience (Lense et al., 2020; Steinberg et al., 2020b).

Given the positive social and emotional effects of musical engagement, along with the prevalence of music use in the home of parents and their young children, it is important to understand the role of everyday musical experiences during the Covid-19 pandemic for parents, their young children, and the parent-child relationship. In the present study, we describe the ways in which parents and their children engage with music during a global pandemic and evaluate if parent musical engagement, child musical engagement, or joint parent-child musical engagement changed as compared to before the Covid-19 pandemic. We then explore the purposes of music use during Covid-19 with an emphasis on the use of music for emotion regulation and social connection. Finally, we consider if parent-child music engagement during Covid-19 relates to the parent-child relationship; specifically, we examine if parent-child music engagement is associated with parents’ affective attachment to their child, i.e., the emotions and thoughts parents have about their child and the parent-child relationship.

## Materials and Methods

### Participants

Participants were 177 caregivers of children between 6 and 70 months old (*M* = 35.9, SD = 17.4), with all ages well-represented throughout the age range. Children were approximately evenly split by gender (48.0% male) and by whether they had a sibling (48.6% only child). Respondents of the survey were primarily the children’s biological mothers (94.4%) and were highly educated (61.7% with a graduate degree). Participants primarily lived in the United States (74%) and Canada (24%). During the Covid-19 pandemic, participants reported losing child care outside of the home, reducing time spent outside of the home, and spending much more time with their children. Full demographics and impact of Covid-19 on caregivers can be found in Table 1.

**TABLE 1:** Sample (*n* = 177) Demographics and Covid-19 Impact.

**Child**	
Sex (% male)	48.0
Age in months [mean (SD)]	35.9 (17.4)
Only child (%)	48.6
**Respondent**	
*Relation to child (%)*	
Biological mother	94.4
*Education level (%)*	
Less than bachelor’s	9.7
Bachelor’s degree	28.6
Graduate degree	61.7
**Prior to Covid-19 (%)**	
Worked outside home	78.5
Childcare outside home	75.7
**During Covid-19**	
Worked outside home (%)	18.1
Childcare outside home (%)	8.5
Reduced or lost wages (%)	7.3
Worked reduced hours (%)	10.2
Did not leave home (%)	61.6
Did not visit friends (%)	83.6
Received a positive Covid-19 test (%)	1.7
*Time spent with child (%)*	
Much more	54.2
Somewhat more	17.0
About the same	20.9
Somewhat less	4.5
Much less	3.4
*Parent distress (K6)*	
Mean (SD)	6.7 (4.3)
Moderate distress (%)	45.8
Severe distress (%)	9.6
*Parenting self-efficacy [mean (SD)]*	4.2 (0.7)
*Parent-child attachment [mean (SD)]*	76.3 (8.3)

### Procedure

Data were collected *via* an online survey using REDCap, a secure, online data server (Harris et al., 2009). The survey assessed demographic information (including impact of Covid-19 on the family), parent and child music engagement and activities, and parent-child attachment, as well as other relevant parent/family characteristics known to impact parent-child attachment such as parent mental health and parenting efficacy. All measures are described below. The survey was available from April to August 2020 and took approximately 20–30 minutes to complete. Over 90% of participants filled out the survey in April or May of 2020 (during the first 2–3 months of lockdown and school/daycare closings in the United States and Canada). The study was approved by the university IRB. Participants provided informed consent prior to accessing the questionnaire.

### Measures

#### Musical Engagement

##### Parent-Child Musical Engagement

Participants responded to an adapted version of the standardized Music@Home scale (Politimou et al., 2018). The measure consisted of 17 items that assessed respondents’ use of music with their child in the home. Participants used a Likert scale ranging from one (completely disagree) to seven (completely agree) to indicate their agreement with statements like “I sing to/with my child several times (e.g., 5–10) a day.” Items were summed for a total Music@Home score, ranging from 17 to 119 (Cronbach’s alpha = 0.92). Items were also divided into two subscales, Child Active Engagement with Music (nine items; Cronbach’s alpha = 0.89) and Parent Initiation of Musical Activities (eight items; Cronbach’s alpha = 0.92).

##### Child Musical Engagement

Participants used a Likert scale ranging from zero (0 min) to six (more than 2 h) to indicate the average amount of time their child spent in various music activities each day. Items included singing, playing instruments, listening to recorded music, playing with musical toys, watching music videos on a TV/tablet, dancing to music, and engaging in music activities with another person.

Participants used a five-point Likert scale ranging from “much less” to “much more” to indicate how much time their child spent watching music videos, listening to recorded music, and engaging in music with another person during Covid-19 as compared to before the pandemic.

Participants used a Likert scale ranging from one (completely disagree) to seven (completely agree) to indicate their agreement with reasons that they or a family member use music in the home with their child. There were 10 possible reasons such as to soothe the child when upset, to get their child’s attention, as part of night-time routines, and to practice communication skills. Responses were re-coded into agree or disagree and then summed for the number of items endorsed to represent a total Function of Music Activities score (Cronbach’s alpha = 0.75).

##### Parent Musical Engagement

Participants used a five-point Likert scale ranging from “much less” to “much more” to indicate how much time they spent listening to and making music during Covid-19 as compared to before the pandemic. Participants also answered Likert scale questions, ranging from one (completely disagree) to seven (completely agree), about how listening to and making music helps them to regulate their emotions, regulate their children’s emotions, socially connect with adults in and outside of their family, and socially connect with their children. Finally, participants indicated how much they now use music to help with their emotion regulation and social connection compared to an average day before Covid-19 using a five-point Likert scale ranging from “much less” to “much more.”

#### Parent-Child Attachment

Parent-child attachment was measured using the Postnatal Attachment Questionnaire (PAQ; Condon and Corkindale, 1998). The PAQ consists of 19 Likert scale items that assess parents’ affective attachment to their child, using questions like “when I am with the child I feel tense and anxious.” Items were summed for a total Parent-Child Attachment score (Cronbach’s alpha = 0.78). The PAQ was originally intended for use with infants but has also been used to assess parent-child attachment in older children (e.g., Goodman and Glenwick, 2012; Steinberg et al., 2020b) because it emphasizes parents’ feelings toward their child, rather than children’s feelings or behaviors toward the parent. We changed the word “baby” to the word “child” in order to make the survey more applicable to our sample.

#### Parent Distress

Parent distress was measured using the Kessler Screening Scale for Psychological Distress (K6; Kessler et al., 2002). The K6 consists of six Likert scale items, ranging from zero (none of the time) to four (all of the time), that assess parents’ mental health through capturing the reported frequency of feelings like nervousness, hopelessness, sadness, and restlessness. Items were summed for a total Parent Distress score (Cronbach’s alpha = 0.83), ranging from zero to 24. A score of zero indicates no distress, one to five indicates low distress, six to 12 indicates moderate distress, and 13–24 indicates high distress (Forman-Hoffman et al., 2014; Tomitaka et al., 2019). Parent distress scores and categorizations for the current sample are provided in Table 1.

#### Parenting Self-Efficacy

Parenting self-efficacy was measured using one question: “Which of the following statements best describes how you feel about yourself as a parent?” Participants responded using a Likert scale ranging from one (not very good at being a parent) to five (a very good parent). This item was adapted from the Longitudinal Study of Australian Children (Zubrick et al., 2008).

#### Non-Musical Parent-Child Activities

To measure overall time spent in parent-child activities, participants responded to the Family Activities Questionnaire (FAQ), adapted from questions used in the Millennium Cohort Study (Hansen et al., 2010). Parents responded to Likert scales ranging from one (hardly ever) to five (several times a day) to describe how frequently they engaged in five different activities with their child (e.g., reading, drawing, playing outdoor games). Items were summed to indicate a total Parent-Child Activity score (Cronbach’s alpha = 0.59).

### Data Analysis

Descriptive statistics were examined for relevant music variables, capturing parent-child musical engagement, child music activities, and parent musical engagement during the Covid-19 pandemic. All five-point Likert items to assess change in music activities during vs. pre-Covid-19 were scaled to range from −2 (much less) to +2 (much more), so that a score of zero reflected a response of “about the same.” We used *t*-tests (one sample, two-tailed) to assess whether parents’ perceptions of music activities/reasons for using music during Covid-19 were significantly different than before Covid-19. Correlations were calculated between parent-child musical engagement, parent-child attachment, parenting efficacy, parent distress, non-musical family activities, parent education, child age, and change in time spent with child during Covid-19. Additionally, we conducted exploratory analyses investigating correlations between particular musical activities, parent reasons for using music with their child, and parent-child attachment.^1^ A linear regression model was used to examine the relationship between parent-child musical engagement and parent-child attachment, controlling for relevant parent variables, change in time parent spent with child due to Covid-19, and child age. Due to the large number of analyses conducted, only *p*-values of <0.01 are reported as significant. All analyses were run in SAS University Edition version 9.4.

## Results

### Musical Experiences

#### Parent-Child Musical Engagement

Parent-child musical engagement during Covid-19 (based on Music@Home total scores) was high across the sample though there was also substantial individual variability in music engagement. Total and subscale scores can be found in Table 2.

**TABLE 2:** Parent-child music engagement and child music activities.

	Mean	SD	Range
**Music@Home**			
Total score	96.6	14.8	60–119
Child active engagement	54.0	7.70	28–63
Parent initiation of music activities	42.6	9.12	14–56
**Child music activities**			
Singing	2.9	1.7	0–6
Playing instruments	2.4	1.4	0–6
Listening to music	3.6	1.6	0–6
Musical toys	2.2	1.5	0–6
Watching musical videos	3.0	1.7	0–6
Dancing to music	2.8	1.6	0–6
Engaging in music socially	3.1	1.5	0–6
**Function of music activities**	7.2	2.5	0–10

*Possible ranges: Music@Home total score: 17–119; child active engagement: 9–63; parent initiation of musical activities: 8–56; child music activities: 0–6; function of music activities: 0–10.*

#### Child Musical Engagement

Parents reported that their child participated in a variety of musical activities (Table 2). Children spent the most time (between 20 and 59 minutes a day) listening to music, engaging in music with another person, and watching music videos. Compared to an average day before Covid-19, parents reported that their child spent significantly more time watching music videos, *t*(173) = 14.10, *p* < 0.0001, *d* = 1.07, listening to recorded music, *t*(173) = 9.37, *p* < 0.0001, *d* = 0.71, and engaging in music with another person, *t*(173) = 8.74, *p* < 0.0001, *d* = 0.66.

Parents endorsed on average between seven to eight (out of 10) reasons for using music with their child. The most popular reason for using music was to keep busy or pass the time while waiting (88.7% endorsed), while the least commonly endorsed reason was to help with transitions between activities (55.9% endorsed). The percent of parents that endorsed each reason can be found in Table 3.

**TABLE 3:** Percent of parents that endorsed using music with their child for each reason.

	Percent
Keep busy/pass the time while waiting	88.7
Interact with child in social games	83.1
Practice academic skills	77.4
Soothe child when upset	76.8
As part of other routines	71.8
Distract child	71.2
As part of nighttime routine	70.6
Practice communication skills	66.7
Get child’s attention	58.8
Help with transitions between activities	55.9

There were small, significant correlations between various child music activities and reasons that parents used music with their children (controlling for child’s age). Activities that involved children’s active participation such as playing instruments, playing with musical toys, dancing to music, and engaging in music socially were generally the most commonly and most strongly correlated with specific functions of musical activities. In contrast, the activity of watching music videos was only associated with the reasons of practicing academic skills, practicing communication skills, and distracting the child. The full correlation table can be found in Supplementary Table 1 in the supplement.

#### Parent Musical Engagement

In regard to their own music engagement, parents reported listening to, *t*(176) = 7.47, *p* < 0.0001, *d* = 0.56, and making, *t*(176) = 2.76, *p* < 0.01, *d* = 0.21, music during Covid-19 significantly more than before the pandemic. Parents agreed or strongly agreed that listening to and making music helps to regulate their emotions, regulate their child’s emotions, and helps them to socially connect with their children (Table 4). They also reported that they were using music for these purposes significantly more during Covid-19 than before the pandemic, *p*′s < 0.0001, *d*′s = 0.51 (regulate own emotions), 0.94 (regulate child’s emotions), 0.99 (socially connect with child). In comparison, compared to before the pandemic, during Covid-19 parents were less likely to use music to socially connect with other adults not in their family, *p* < 0.001, *d* = −0.28. There were no changes in using music to connect with adults in their family during the pandemic.

**TABLE 4:** Parent reports of the purpose of listening to or making music during Covid-19.

	Agreement^a^		Covid-19 change^b^	
		
	*M*	SD	*M*	SD
Regulate my emotions/change mood or physical state	5.78	1.05	0.43***	0.84
Regulate my child’s emotions	5.66	0.97	0.64***	0.68
Socially connect with other adults not in my family	4.49	1.29	−0.30**	1.07
Socially connect with other adults in my family	4.90	1.26	0.16	0.90
Socially connect with my child/children	5.82	0.89	0.79***	0.80

^*a*^Agreement: Parents expressed their agreement with each reason on a scale of 1 (completely disagree) to 7 (completely agree). ^*b*^Covid-19 change: Parents expressed how much they currently used music for each reason compared to an average day before Covid-19 where −2 = much less, −1 = somewhat less, 0 = about the same, 1 = somewhat more, and 2 = much more. ** *p* < 0.001 and *** *p* < 0.0001.

### Correlations Between Parent-Child Attachment and Other Variables

There was a significant, small, positive correlation between parent-child musical engagement and parent-child attachment, *r* = 0.29, *p* < 0.001. Parent-child attachment was also significantly correlated with parent distress, *r* = −0.40, *p* < 0.001, parenting self-efficacy, *r* = 0.50, *p* < 0.001, and parent education, *r* = −0.27, *p* < 0.001. Parent-child musical engagement was significantly correlated with non-musical family activities, *r* = 0.23, *p* < 0.01. The full correlation matrix can be found in Table 5.

**TABLE 5:** Correlations between parent-child attachment, parent-child musical engagement, and other variables.

	**1.**	**2.**	**3.**	**4.**	**5.**	**6.**	**7.**
1. Attachment							
2. Music@Home	0.29***						
3. Parent distress	−0.40***	–0.11					
4. Parenting self-efficacy	0.50***	0.10	−0.32***				
5. Parent education	−0.27**	–0.05	0.04	0.02			
6. Child age	–0.16	–0.11	–0.16	–0.07	0.11		
7. Non-musical family activities	0.16	0.23*	–0.18	0.10	0.14	0.16	
8. Time spent with child during Covid-19	–0.14	0.11	–0.09	0.02	0.29***	0.19	0.17

*p < 0.01, **p < 0.001, and ***p < 0.0001.

### Parent-Child Attachment and Parent-Child Musical Engagement

The model including Music@Home total score, parent distress and self-efficacy, parent education level, child age, non-musical family activities, and change in time spent with child during Covid-19 significantly related to parent-child attachment scores, *F*(7, 165) = 18.61, *p* < 0.0001. While the largest predictors of parent-child attachment were parent-related variables of parent distress, self-efficacy, and parent education, parent-child music (See Table 6 for the full parent-child attachment model) engagement was still significantly associated with parent-child attachment even when controlling for these variables. In contrast, non-musical family activities was not a significant predictor.

**TABLE 6:** Parent-child attachment model.

	**β**	***t***	**$\eta_{p}^{2}$**
Intercept	62.04	10.10***	
Music@Home total score	0.10	2.97*	0.051
Parent distress	–0.50	−4.15***	0.094
Parenting self-efficacy	4.21	5.98***	0.178
Parent education	–1.51	−3.69**	0.076
Child age	–0.06	–2.08	0.025
Non-musical family activities	0.27	1.57	0.015
Time spent with child during Covid-19	–0.77	–1.67	0.017

*R*^2^ = 0.44. * *p* < 0.01, ** *p* < 0.001, and *** *p* < 0.0001.

To further unpack how specific music activities relate to parent-child attachment, we also conducted exploratory correlations between parent-child attachment and the two Music@Home subscales, as well as between parent-child attachment, specific child music activities, and specific reasons why parents used music with their child (controlling for other variables significantly associated with attachment and child age). There were significant small-to-moderate correlations with several items, notably the Music@Home Parent Initiation of Music subscale (*r* = 0.26, *p* < 0.001), three of the child music behavior items (playing instruments, *r* = 0.31, *p* < 0.001; dancing to music, *r* = 0.29, *p* < 0.001; engaging in music socially, *r* = 0.29, *p* < 0.001), and several of the reasons for using music. See Supplementary Table 2 in the Supplement for all correlations.

## Discussion

The Covid-19 pandemic considerably altered the lives of parents of young children. Disruptions to everyday life, including loss of childcare (Patrick et al., 2020) and changes in employment situations (Craig and Churchill, 2020) were common across the world as a result of mandated lockdowns and stay at home orders, with parents of young children experiencing increased stress levels and time spent with their children (Calarco et al., 2020; Russell et al., 2020). Given the high prevalence of parent-child musical engagement in the home (Politimou et al., 2018) and the known benefits of music for emotion regulation and social connection (Freeman, 2000; Dunbar, 2012; Moore, 2013), the current study aimed to understand how parents and children engaged in music activities during the pandemic. Results demonstrated that (1) parents frequently engaged in musical activities with their young children during the Covid-19 pandemic; (2) parents perceived that their own and their child’s music activities increased during Covid-19; (3) parents reported using music for both their own and their child’s emotion regulation, as well as for social connection with their children during Covid-19; and (4) parent-child music engagement significantly predicted parent-child attachment, controlling for relevant parent variables, including parent distress, efficacy, and education, parent-child engagement in non-musical activities, child age, and change in time spent with their child during Covid-19.

Parents frequently engaged in musical activities with their child during the Covid-19 pandemic. Even though life and routines drastically changed for families during the pandemic, parent-child music activities remained a part of parent-child interactions consistent with the ubiquity of parent-child musical engagement during non-pandemic times (Steinberg et al., 2020b). In fact, parents reported that their child’s, their own, and their joint engagement with music increased overall during the pandemic; effect sizes for these perceptions of changes in music activities were medium to large. Parents perceived that their child listened to music, watched music videos, and engaged in music with another person significantly more than they did before the pandemic. Listening to music and watching music videos (passive musical activities) and engaging in music socially (an active, shared musical activity) were the activities that parents reported children spent the most time doing, perhaps reflecting both parents’ need to keep children busy alone while parents attended to other matters and their increased time with their child. Indeed, parents reported using music for specific functional purposes with their child, which aligned with the specific types of musical activities. These findings are similar to those of other music questionnaire studies, which suggested that listening, singing, and dancing to music with others are common activities for children under 5 years of age (Custodero et al., 2003; Blackburn, 2017); families appear to continue to utilize these activities, perhaps at an increased level, during Covid-19.

There was a strong consensus (means that fell between agree and strongly agree) that parents listened to and/or made music for their own and their children’s emotion regulation and in order to socially connect with their children. Indeed, effect sizes were strong for parents using music as a tool for/with their children’s emotional and social needs vs. parents using music to connect with other adults (within or outside of their household). This may reflect the increased time parents were spending with their child due to Covid-19, the importance of parents in supporting children’s social-emotional needs, and/or the general centrality of the parent-child relationship for parents of young children (Borkowski et al., 2001). Music has been linked to several positive psychosocial outcomes in regard to emotions, relationships, and mood including for parents of young children (Hargreaves and North, 1999; Custodero et al., 2003; Croom, 2015; Fancourt and Perkins, 2017). Our results indicate that during a global pandemic, music continues to serve as a social and emotional tool for parents and children.

Consistent with this, parent-child musical engagement was positively related to parent-child attachment, even when controlling for high parent distress levels during Covid-19 and parenting efficacy and education, constructs that are highly related to parent-child attachment (Atkinson et al., 2000; Kohlhoff and Barnett, 2013). In contrast, spending time with children in non-musical activities did not significantly relate to parent-child attachment. When examining the two Music@Home subscales separately, the Parent Initiation of Music subscale was particularly associated with parent-child attachment. This may reflect the importance of parents being actively engaged in an activity with their child and/or their intentionality in sharing meaningful, social experiences with their child (note that the Postnatal Attachment Questionnaire used in the present study reflects *parents’* affective attachment to their child). Consistent with this, exploratory analyses revealed that the music activities most associated with parent-child attachment were ones which afforded opportunities for parents and children to have productive, active roles in a social context such as dancing or engaging in social games. The relationship between interpersonal, social music activities with parents’ affective attachment is also consistent with broader theories of musicality emerging because it supports social bonding (Savage et al., 2020).

Although not causal, the positive relationship between parent-child musical activities and parent-child attachment suggests that joint musical experiences may be a contributor to facilitating parents’ affective connection to their child even during the increased stress, increased parenting time, and other life changes occurring in the midst of a global pandemic. Due to the cross-sectionality of the current study, the direction of the findings cannot be tested, and it may be that parents who are close to their children engage in more musical activities (though note no relationship was observed between parent-child attachment and non-musical activities). Of course, other family, child, and parent factors and activities which were not captured in the current study also contribute to parent-child attachment during Covid-19.

As parent and child stress, mental health difficulties, and parent-child conflict are increased during Covid-19 (Russell et al., 2020), it is essential to consider activities that support parent-child attachment, which is linked with both parent and child well-being (Johnson, 2013; de Cock, Evi et al., 2016). Previous research with parents of children under 12 years old found that activities like playing games and watching TV with their child increased the most during the Covid-19 pandemic, with less than half (40.1%) of their participants reporting an increase in parent-child singing (Lee et al., 2020). As the current study indicates that music in particular is beneficial for supporting the parent-child relationship, future work can consider how to support parents in incorporating musical activities into their home routines during times of increased caregiving responsibilities and stress.

Another limitation of the current study to be addressed in future studies was the high education level of participants (61.7% with a graduate degree), which may have contributed to parent reports of frequent musical activities (e.g., Custodero et al., 2003). While we controlled for parent education in the parent-child attachment model, a more representative sample could expand our knowledge of music in the home across diverse families and situations. At the onset of Covid-19, highly educated professionals had the most flexibility to work remotely from home (Dey et al., 2020). Likewise, our participants exhibited this flexibility: 78.5% of participants worked outside the home before the pandemic, while 18.1% worked outside the home during the pandemic, with only 7.3% of participants reporting reduced or lost wages. Thus, while music and parent-child attachment were related for this group of parents, the relationship may differ for parents experiencing more adverse direct effects of Covid-19, like losing employment. At the same time, our sample did exhibit elevated levels of parent distress, with 45.8% of parents experiencing moderate levels of distress and 9.6% of parents experiencing severe levels of distress. Comparatively, in 2018, only 21.4% of a national sample of adults with children under 18 years old in their home exhibited moderate or severe levels of parent distress (Twenge and Joiner, 2020). Given the use of an online survey to collect data, parents with internet access and time to complete the survey were the most likely to fill it out. As such, the sample may have spent more time with their children during the pandemic than the general parent population, and had more time to engage in musical (and other) activities. Additionally, the majority of the present data was collected in the first months of the pandemic-associated closures and lockdowns in the United States and Canada. Longitudinal studies could assess how use of music and its impact on the parent-child relationship changes with prolonged experience of the pandemic. Finally, for a subset of items, participants were asked to retrospectively compare their purposes for using music before the pandemic to their current situation, which may be subject to response bias.

Music is a powerful tool for bringing parents and their children together. Amidst changed routines and high stress of the Covid-19 pandemic, music was a tool for emotion regulation and a way for parents to socially connect with their children; parents’ scores on the standardized Music@Home survey significantly related to their affective attachment toward their child. Findings suggest that even during times of increased caregiving needs and high parent distress, music continues to be a ubiquitous part of families’ lives and may play a role in and/or reflect the parent-child relationship.

## Data Availability Statement

The raw data supporting the conclusions of this article will be made available by the authors, without undue reservation.

## Ethics Statement

The studies involving human participants were reviewed and approved by Vanderbilt University Institutional Review Board. The participants provided their written informed consent to participate in this study.

## Author Contributions

ML designed the study. SS and TL cleaned the data. SS analyzed the data. SS, TL, and ML contributed to the manuscript. All authors contributed to the article and approved the submitted version.

## Conflict of Interest

The authors declare that the research was conducted in the absence of any commercial or financial relationships that could be construed as a potential conflict of interest.

## Footnotes

1 We thank our reviewers for this suggestion.

## References

[B1] Altig D. Baker S. Barrero J. M. Bloom N. Bunn P. Chen S. (2020). Economic uncertainty before and during the COVID-19 pandemic. *J. Public Econom.* 191:104274. 10.1016/j.jpubeco.2020.104274 PMC7480328 32921841

[B2] Atkinson L. Paglia A. Coolbear J. Niccols A. Parker K. C. Guger S. (2000). Attachment security: A meta-analysis of maternal mental health correlates. *Clin. Psychol. Rev.* 20 1019–1040. 10.1016/s0272-7358(99)00023-9 11098398

[B3] Bartik A. W. Cullen Z. B. Glaeser E. L. Luca M. Stanton C. T. (2020). What jobs are being done at home during the COVID-19 crisis? Evidence from firm-level surveys. *Natl. Bur. Econom. Res.*, NBER Working Paper No. 27422; JEL No. J01, J24, M5, O3. 1–16.

[B4] Blackburn C. (2017). Young children’s musical activities in the home. *Education* 45 674–688. 10.1080/03004279.2017.1342320

[B5] Borkowski J. G. Ramey S. L. Bristol-Power M. (Eds.). (2001). *Parenting and the Child’s World: Influences on Academic, Intellectual, and Social-Emotional Development.* Hove: Psychology Press.

[B6] Bulfone T. Quattrin R. Zanotti R. Regattin L. Brusaferro S. (2009). Effectiveness of music therapy for anxiety reduction in women with breast cancer in chemotherapy treatment. *Holist Nurs. Pract.* 23 238–242. 10.1097/hnp.0b013e3181aeceee 19574761

[B7] Calarco J. M. Anderson E. Meanwell E. V. Knopf A. (2020). *“Let’s not Pretend it’s Fun”: How COVID-19-Related School and Childcare Closures are Damaging Mothers’ Well-Being.* Available online at: 10.31235/osf.io/jyvk4 (accessed November 25, 2020).

[B8] Cirelli L. K. Einarson K. M. Trainor L. J. (2014). Interpersonal synchrony increases prosocial behavior in infants. *Dev. Sci.* 17 1003–1011. 10.1111/desc.12193 25513669

[B9] Cirelli L. K. Jurewicz Z. B. Trehub S. E. (2020). Effects of maternal singing style on mother–infant arousal and behavior. *J. Cogn. Neurosci.* 32 1213–1220. 10.1162/jocn_a_01402 30912725

[B10] Cirelli L. K. Trehub S. E. Trainor L. J. (2018). Rhythm and melody as social signals for infants. *Annal. N. Y. Acad. Sci.* 1423 66–72. 10.1111/nyas.13580 29512877

[B11] Condon J. T. Corkindale C. J. (1998). The assessment of parent-to-infant attachment: development of a self-report questionnaire instrument. *J. Reproduct. Infant Psychol.* 16 57–76. 10.1080/02646839808404558

[B12] Craig L. Churchill B. (2020). Dual-earner parent couples’ work and care during COVID−19. *Gender Work Organiz.* 28 66–79. 10.1111/gwao.12497 32837023 PMC7362071

[B13] Croom A. M. (2015). Music practice and participation for psychological well-being: a review of how music influences positive emotion, engagement, relationships, meaning, and accomplishment. *Musicae Sci.* 19 44–64. 10.1177/1029864914561709

[B14] Custodero L. A. Johnson-Green E. A. (2008). Caregiving in counterpoint: reciprocal influences in the musical parenting of younger and older infants. *Early Child Dev. Care* 178 15–39. 10.1080/03004430600601115

[B15] Custodero L. A. Rebello Britto P. Brooks-Gunn J. (2003). Musical lives: a collective portrait of American parents and their young children. *J. Appl. Dev. Psychol.* 24 553–572. 10.1016/j.appdev.2003.08.005

[B16] Czeisler M. É Lane R. I Petrosky E. Wiley J. F. Christensen A. Njai R. (2020). Mental health, substance use, and suicidal ideation during the COVID-19 pandemic — United States, June 24–30, 2020. *Morb. Mortal. Weekly Rep.* 69 1049–1057. 10.15585/mmwr.mm6932a1 32790653 PMC7440121

[B17] de Cock Evi S. A. Henrichs J. Vreeswijk C. M. J. M. Maas A. J. Rijk C. H. A. M. (2016). Continuous feelings of love? The parental bond from pregnancy to toddlerhood. *J. Fam. Psychol.* 30 125–134. 10.1037/fam0000138 26280095

[B18] de Vries P. (2009). Music at home with the under fives: what is happening? *Early Child Dev. Care* 179 395–405. 10.1080/03004430802691914

[B19] Del Boca D. Oggero N. Profeta P. Rossi M. (2020). *Women’s Work, Housework and Childcare, Before and During COVID-19*. IZA Discussion Paper No. 13409. Bonn: Institute of Labor Economics (IZA). 10.1007/s11150-020-09502-1 PMC7474798 32922242

[B20] Dey M. Frazis H. Loewenstein M. Sun H. (2020). Ability to work from home: evidence from two surveys and implications for the labor market in the COVID-19 pandemic. *Month. Lab. Rev.* 2 1–19.

[B21] Dunbar R. I. M. (2012). Bridging the bonding gap: the transition from primates to humans. *Philos. Trans. R. Soc. Lond. B Biol. Sci.* 367 1837–1846. 10.1098/rstb.2011.0217 22641822 PMC3367699

[B22] Erkkilä J. Punkanen M. Fachner J. Ala-Ruona E. Pöntiö I. Tervaniemi M. (2011). Individual music therapy for depression: randomised controlled trial. *Br. J. Psychiatry* 199 132–139. 21474494 10.1192/bjp.bp.110.085431

[B23] Fancourt D. Perkins R. (2017). Associations between singing to babies and symptoms of postnatal depression, wellbeing, self-esteem and mother-infant bond. *Public Health* 145 149–152. 10.1016/j.puhe.2017.01.016 28359384

[B24] Fancourt D. Perkins R. (2018a). Effect of singing interventions on symptoms of postnatal depression: three-arm randomised controlled trial. *Br. J. Psychiatry* 212 119–121. 10.1192/bjp.2017.29 29436333

[B25] Fancourt D. Perkins R. (2018b). The effects of mother–infant singing on emotional closeness, affect, anxiety, and stress hormones. *Music Sci.* 1 1–10. 10.1177/2059204317745746

[B26] Forman-Hoffman V. L. Muhuri P. K. Novak S. P. Pemberton M. R. Ault K. L. Mannix D. (2014). *Psychological Distress and Mortality Among Adults in the US Household Population. CBHSQ Data Review.* Rockville, MD: SAMHSA.

[B27] Freeman W. J. III (2000). “A neurobiological role of music in social bonding,” in *The Origins of Music*, eds Wallin N. Merkur B. Brown S. (Cambridge, MA: MIT Press), 411–424.

[B28] Gassman-Pines A. Ananat E. O. Fitz-Henley J. (2020). COVID-19 and parent-child psychological well-being. *Pediatrics* 146 1–9. 10.1542/peds.2020-007294 PMC7546085 32764151

[B29] Goodman S. J. Glenwick D. S. (2012). Correlates of attachment perceptions in parents of children with Autism Spectrum Disorders. *J. Aut. Dev. Disord.* 42 2056–2066. 10.1007/s10803-012-1453-8 22298107

[B30] Hansen K. Jones E. Joshi H. Budge D. (2010). *Millennium Cohort Study Fourth Survey: A User’s Guide to Initial Findings.* London: Centre for Longitudinal Studies, University of London.

[B31] Hanser S. B. Thompson L. W. (1994). Effects of a music therapy strategy on depressed older adults. *J. Gerontol.* 49 265–269. 10.1093/geronj/49.6.p265 7963281

[B32] Hargreaves D. J. North A. C. (1999). The functions of music in everyday life: redefining the social in music psychology. *Psychol. Music* 27 71–83. 10.1177/0305735699271007

[B33] Harris P. A. Taylor R. Thielke R. Payne J. Gonzalez N. Conde J. G. (2009). Research electronic data capture (REDCap)—a metadata-driven methodology and workflow process for providing translational research informatics support. *J. Biomed. Inform.* 42 377–381. 10.1016/j.jbi.2008.08.010 18929686 PMC2700030

[B34] Ilari B. (2005). On musical parenting of young children: musical beliefs and behaviors of mothers and infants. *Early Child Dev. Care* 175 647–660. 10.1080/0300443042000302573

[B35] Johnson K. (2013). Maternal-infant bonding: a review of literature. *Int. J. Childb. Educ.* 28 17–22.

[B36] Kessler R. C. Andrews G. Colpe L. J. Hiripi E. Mroczek D. K. Normand S. L. (2002). Short screening scales to monitor population prevalences and trends in non-specific psychological distress. *Psychol. Med.* 32 959–976. 10.1017/s0033291702006074 12214795

[B37] Kirschner S. Tomasello M. (2010). Joint music making promotes prosocial behavior in 4-year-old children. *Evol. Hum. Behav.* 31 354–364. 10.1016/j.evolhumbehav.2010.04.004

[B38] Kohlhoff J. Barnett B. (2013). Parenting self-efficacy: links with maternal depression, infant behaviour and adult attachment. *Early Hum. Dev.* 89 249–256. 10.1016/j.earlhumdev.2013.01.008 23398731

[B39] Kreutz G. Murcia C. Q. Bongard S. (2012). “Psychoneuroendocrine research on music and health: an overview,” in *Music, Health, and Wellbeing*, eds MacDonald R. Kreutz G. Mitchell L. (Oxford: Oxford University Press), 457–476.

[B40] Lee S. J. Ward K. P. Chang O. D. Downing K. M. (2020). Parenting activities and the transition to home-based education during the COVID-19 pandemic. *Child. Youth Serv. Rev.* 2:105585. 10.1016/j.childyouth.2020.105585 33071407 PMC7553006

[B41] Lense M. D. Beck S. Liu C. Pfeiffer R. Diaz N. Lynch M. (2020). Parents, peers, and musical play: Integrated parent-child music class program supports community participation and well-being for families of children with and without autism spectrum disorder. *Front. Psychol.* 11:2775. 10.3389/fpsyg.2020.555717 33192810 PMC7662132

[B42] Lense M. D. Jones W. R. (2016). “Beat-based entrainment during infant-directed singing supports social engagement,” in *Proceedings of the 14th International Conference on Music Perception & Cognition (icmpc14)*, San Francisco, CA.

[B43] Linnemann A. Ditzen B. Strahler J. Doerr J. M. Nater U. M. (2015). Music listening as a means of stress reduction in daily life. *Psychoneuroendocrinology* 60 82–90. 10.1016/j.psyneuen.2015.06.008 26142566

[B44] Mehr S. A. Song L. A. Spelke E. S. (2016). For 5-month-old infants, melodies are social. *Psychol. Sci.* 27 486–501. 10.1177/0956797615626691 26917211

[B45] Mendoza J. K. Fausey C. M. (2019). Everyday music in infancy. *PsyArXiv* [Preprint]. 10.31234/osf.io/sqatb PMC8596421 34170059

[B46] Moore K. S. (2013). A systematic review on the neural effects of music on emotion regulation: implications for music therapy practice. *J. Music Ther.* 50 198–242. 10.1093/jmt/50.3.198 24568004

[B47] Nicholson J. M. Berthelsen D. Abad V. Williams K. Bradley J. (2008). Impact of music therapy to promote positive parenting and child development. *J. Health Psychol.* 13 226–238. 10.1177/1359105307086705 18375628

[B48] Patrick S. W. Henkhaus L. E. Zickafoose J. S. Lovell K. Halvorson A. Loch S. (2020). Well-being of parents and children during the COVID-19 pandemic: a national survey. *Pediatrics* 146 1–9. 10.1542/peds.2020-016824 32709738

[B49] Perkins R. Yorke S. Fancourt D. (2018). How group singing facilitates recovery from the symptoms of postnatal depression: a comparative qualitative study. *BMC Psychol.* 6:41. 10.1186/s40359-018-0253-0 30119704 PMC6098577

[B50] Persico G. Antolini L. Vergani P. Costantini W. Nardi M. T. Bellotti L. (2017). Maternal singing of lullabies during pregnancy and after birth: Effects on mother–infant bonding and on newborns’ behaviour. Concurrent Cohort Study. *Women Birth* 30 e214–e220. 28169158 10.1016/j.wombi.2017.01.007

[B51] Politimou N. Stewart L. Müllensiefen D. Franco F. (2018). Music@Home: a novel instrument to assess the home musical environment in the early years. *PLoS One* 13:e0193819. 10.1371/journal.pone.0193819 29641607 PMC5894980

[B52] Russell B. S. Hutchison M. Tambling R. Tomkunas A. J. Horton A. L. (2020). Initial challenges of caregiving during COVID-19: caregiver burden, mental health, and the parent–child relationship. *Child Psychiatry Hum. Dev.* 51 671–682. 10.1007/s10578-020-01037-x 32749568 PMC7398861

[B53] Savage P. Loui P. Tarr B. Schachner A. Glowacki L. Mithen S. (2020). Music as a coevolved system for social bonding. *Behav. Brain Sci.* 1–36. 10.1017/S0140525X20000333 [Epub ahead of print]. 32814608

[B54] Steinberg S. Liu T. Lense M. D. (2020a). Musical engagement and parent-child attachment in families with young children during the Covid-19 pandemic. *PsyArXiv* [Preprint]. 10.31234/osf.io/v458u PMC8019693 33828508

[B55] Steinberg S. Shivers C. M. Liu T. Cirelli L. K. Lense M. D. (2020b). Musical engagement and parent-child attachment in families of young children with and without developmental disabilities. *PsyArXiv* [Preprint]. 10.31234/osf.io/xveqy

[B56] Tarr B. Launay J. Dunbar R. I. (2014). Music and social bonding:“self-other” merging and neurohormonal mechanisms. *Front. Psychol.* 5:1096. 10.3389/fpsyg.2014.01096 PMC4179700 25324805

[B57] Thayer R. E. Newman J. R. McClain T. M. (1994). Self-regulation of mood: Strategies for changing a bad mood, raising energy, and reducing tension. *J. Pers. Soc. Psychol.* 67 910–925. 10.1037/0022-3514.67.5.910 7983582

[B58] Thoma M. V. Ryf S. Mohiyeddini C. Ehlert U. Nater U. M. (2012). Emotion regulation through listening to music in everyday situations. *Cogn. Emot.* 26 550–560. 10.1080/02699931.2011.595390 21902567

[B59] Tomitaka S. Kawasaki Y. Ide K. Akutagawa M. Ono Y. Furukawa T. A. (2019). Distribution of psychological distress is stable in recent decades and follows an exponential pattern in the US population. *Sci. Rep.* 9 1–10. 31427587 10.1038/s41598-019-47322-1 PMC6700099

[B60] Trehub S. E. Plantinga J. Russo F. A. (2016). Maternal vocal interactions with infants: reciprocal visual influences. *Soc. Dev.* 25 665–683. 10.1111/sode.12164

[B61] Twenge J. Joiner T. (2020). Mental distress among US adults during the COVID-19 pandemic. *Clin. Psychol.* 76 2170–2182. 10.31234/osf.io/wc8ud PMC7675251 33037608

[B62] U.S. Chamber of Commerce Foundation (2020). *Childcare: An Essential Industry for Economic Recovery. Childcare Providers and Covid-19, 3, 1-21.* Washington, DC: U.S. Chamber of Commerce Foundation.

[B63] Von Lob G. Camic P. Clift S. (2010). The use of singing in a group as a response to adverse life events. *Int. J. Ment. Health Promot.* 12 45–53. 10.1080/14623730.2010.9721818

[B64] Yehuda N. (2011). Music and stress. *J. Adult Dev.* 18 85–94. 10.1007/s10804-010-9117-4

[B65] Zubrick S. R. Smith G. J. Nicholson J. Sanson A. Jackiewicz T. A. (2008). *Parenting and Families in Australia. FaHCSIA Social Policy Research Paper.* Available online at: https://ssrn.com/abstract=1703269 (accessed June 1, 2008).

