# Supplementary material for: Musical Engagement and Parent-Child Attachment in Families With Young Children During the Covid-19 Pandemic
Source: Front Psychol. 2021 Mar 22;12:641733. doi: 10.3389/fpsyg.2021.641733 (PMC8019693; doi:10.3389/fpsyg.2021.641733)
Supplement: Supplementary file 1 [file Table_1.pdf]

Supplement

Table 1S

Correlations between child music activities and parent reasons for using music with their child

	Sing	Play	Listen	Toys	Watch	Dance	Engage
Keep busy/pass the time while waiting	0.13	0.20*	0.22*	0.24*	0.10	0.22*	0.36***
Interact with child in social games	0.27**	0.30***	0.34***	0.35***	0.15	0.35***	0.45***
Practice academic skills	0.20*	0.31***	0.35***	0.34***	0.26**	0.34***	0.32***
Soothe child when upset	0.18	0.24*	0.27**	0.20*	0.14	0.31***	0.43***
As part of other routines	0.21*	0.28**	0.19	0.16	0.17	0.32***	0.37***
Distract child	0.12	0.26**	0.16	0.30***	0.24*	0.27**	0.35***
As part of nighttime routine	0.12	0.18	0.16	0.06	-0.02	0.20*	0.25**
Practice communication skills	0.19	0.31***	0.15	0.28**	0.24*	0.25**	0.28**
Get child's attention	0.14	0.28**	0.06	0.32***	0.16	0.28**	0.32***
Help with transitions between activities	0.06	0.17*	0.22*	0.28***	0.09	0.28**	0.30***

Note. All correlations control for child age

*$p < 0.01$ **$p < 0.001$ ***$p < 0.0001$

Table 2S

Correlations between parent-child attachment and Music@Home Subscales, child music activities, and parent reasons for using music with their child

	Parent-Child Attachment
Music@Home	
Child Active Engagement	0.16
Parent Initiation of Music Activities	0.26**
Child Music Activities	
Singing	0.15
Playing instruments	0.31***
Listening to music	0.19
Musical toys	0.13
Watching musical videos	-0.02
Dancing to music	0.29**
Engaging in music socially	0.29***
Parent Reasons for Using Music	
Keep busy/pass the time while waiting	0.24*
Interact with child in social games	0.21*
Practice academic skills	0.20*
Soothe child when upset	0.21*
As part of other routines	0.19
Distract child	0.13
As part of nighttime routine	0.08
Practice communication skills	0.13
Get child's attention	0.22*
Help with transitions between activities	0.17

Note. All correlations control for child age, parent education, parent distress, and parenting self-efficacy

*$p < 0.01$ **$p < 0.001$ ***$p < 0.0001$
